# The G1/S repressor *WHI5* is expressed at similar levels throughout the cell cycle

**DOI:** 10.1186/s13104-022-06142-9

**Published:** 2022-07-15

**Authors:** Sylvain Tollis

**Affiliations:** Institute of Biomedicine, University of Eastern Finland, Kuopio, Finland

**Keywords:** Whi5 dilution model, Cell cycle, Cell size, Cell growth, G1/S transcription, G1/S repression, Confocal microscopy, Single mRNA imaging

## Abstract

**Objectives:**

While it is clear that cells need to grow before committing to division at the G1/S transition of the cell cycle, how cells sense their growth rate or size at the molecular level is unknown. It has been proposed that, in budding yeast, the dilution of the Whi5 G1/S transcriptional repressor as cells grow in G1 is the main driver of G1/S commitment. This model implies that Whi5 synthesis is substantially reduced in G1 phase. Recent work has reported that the concentration of Whi5 is size- and time-independent in G1 cells, challenging the dilution model. These results in turn imply that Whi5 must be synthesized in G1 phase, but the cell cycle dependence of *WHI5* mRNA expression has not been examined in live cells.

**Results description:**

To address this question, we monitored single *WHI5* mRNA molecules in single live cells using confocal microscopy, and quantified *WHI5* mRNA copy number in G1, G1/S, and S/G2/M phase cells. We observed that *WHI5* mRNA is found in very similar amount irrespective of cell cycle stage. The constant *WHI5* mRNA copy number throughout G1 phase rules out alterations in mRNA abundance as a contributing factor for any putative dilution of Whi5.

**Supplementary Information:**

The online version contains supplementary material available at 10.1186/s13104-022-06142-9.

## Introduction

How the decision to divide is linked to volumetric cell growth is still unclear. A certain amount of growth is required from birth before cells can commit to division, however how cells sense their growth/size at the molecular level is unknown. Yeast cells commit to division (“Start”) at the end of G1 phase [1, 2] where they initiate a complex transcriptional program of ~ 200 genes that encode proteins necessary for e.g. bud emergence and DNA replication. This program is controlled in part by the Swi4/6 Cell Cycle Box (or SCB-) binding factor (SBF) transcription factor [3, 4]. In G1, SBF is inhibited by the Whi5 transcriptional repressor [5–7]. Hence, Start activation requires reduction of Whi5 repressive activity [6–8], and a number of different molecular mechanisms has been proposed to explain this early cell cycle event [9–17]. In particular it was proposed by Schmoller et al. [12] that as the nucleus grows during G1, the nuclear concentration of Whi5 slowly decreases, thereby increasing the probability of the Start transition. This model of passive Whi5 dilution requires that Whi5 protein synthesis substantially drops in G1. Recent works have reported size- and time-independent Whi5 protein concentration in G1 cells [10, 11, 14, 18], challenging the dilution model. These results suggest that Whi5 protein is not substantially diluted as cells grow, and therefore that Whi5 synthesis continues in G1. In turn, these data imply that *WHI5* mRNA should still be expressed in G1 cells.

The current work extends on [18] and aims to address the cell cycle dependence of *WHI5* transcription. *WHI5* mRNA abundance as a function of cell size has been reported using bulk RNA-sequencing of Fluorescence-Activated Cell Sorting (FACS)-sorted size fractions, and in single fixed cells using single molecule Fluorescence In-Situ Hydridization (smFISH, [19]), and as a function of the cell cycle using smFISH [20]. While no size-dependence of *WHI5* mRNA was reported in the former study, the latter reported slightly more elevated levels in S/G2/M cells. This modest discrepancy could be attributable to mRNA degradation during fixation/treatments required for smFISH [21], in particular since *WHI5* mRNA half-life is of the order of 1 min [22].

Here, we used live cell microscopy to monitor *WHI5* mRNAs in single live cells without the need for synchronization or fixation/treatments methods that may alter the abundance of unstable mRNAs. We quantified *WHI5* mRNA copy number in G1, G1/S, and S/G2/M cells. We found *WHI5* mRNA in very similar amount irrespective of the cell cycle stage. These results demonstrate that *WHI5* is actively expressed in G1 cells, and provide a mechanism whereby cells overcome growth-dependent passive dilution.

## Main text

To monitor single *WHI5* mRNA molecules we have used the PP7 tagging system [23]. Briefly, we inserted 12 copies of a RNA hairpin at the Whi5 C-terminal of wild type (WT) BY4741 yeasts (see Methods for details). The resulting *whi5::WHI5-12xPP7hairpin-KanMX* strain will be referred to as Whi5-PP7tag for brevity. The hairpin is recognized with high affinity and specificity by the PP7 bacteriophage coat protein. We transformed WT and Whi5-PP7tag cells with a plasmid constitutively expressing a 2x-yeGFP-PP7 coat protein fusion expressed from the moderate level ADE3 promoter. In the former control strain, yeGFP forms natural dimers but does not cluster, while in the latter strain yeGFP dimers avidly bind to the Whi5-12xPP7 hairpin tag, hence instantaneously labeling each single *WHI5* mRNA molecule with 24-yeGFPs that form a bright spot [23]. These strains are referred to as yeGFP and Whi5mRNA-24x-yeGFP respectively.

**Fig. 1:**
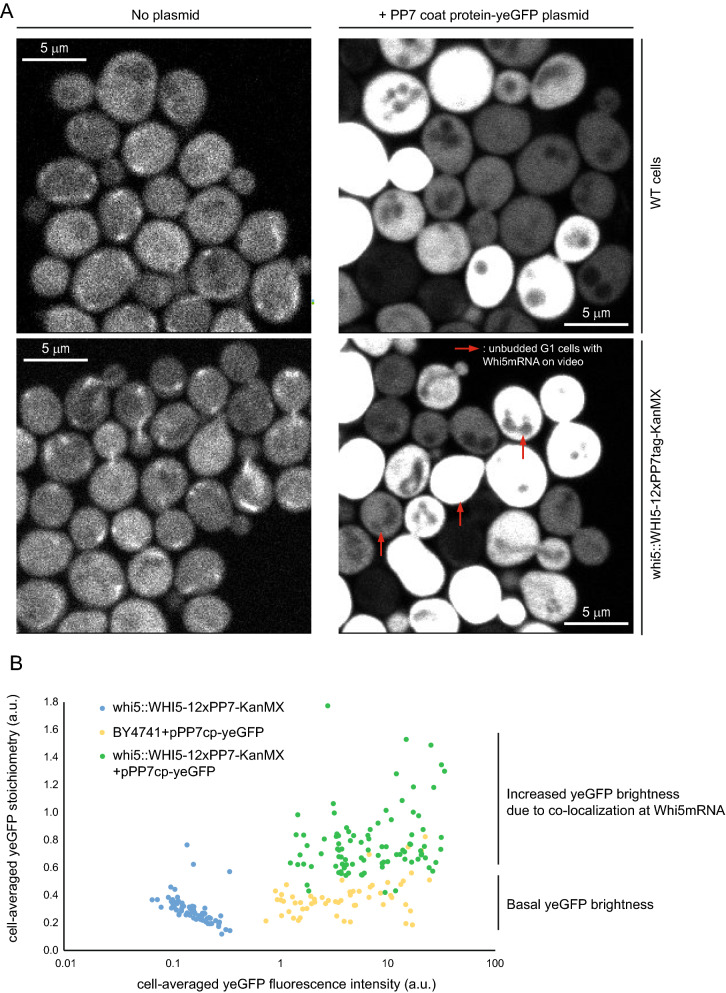
*WHI5* mRNA is detected across all cell cycle stages. **A** Projections of the sN&B time-series of the same cells shown on Additional file 2: Video S1, Additional file 3: Video S2, Additional file 4: Video S3, Additional file 5: 4 Video S4, using a different intensity scale allowing for the visualization of cells from auto-fluorescence only in absence of 2x-yeGFP plasmid. Red arrows indicate exemplary unbudded G1 cells showing clear *WHI5* mRNA spots in Whi5mRNA-24x-yeGFP cells exclusively. **B** Cell-averaged yeGFP stoichiometry (vertical axis) as a function of cell-averaged yeGFP fluorescence intensity (horizontal axis) in individual single yeGFP cells (yellow dots), Whi5-PP7tag cells (blue dots) and Whi5mRNA-24x-yeGFP cells (green dots). Each dot represents an individual cell. yeGFP stoichiometry was identified to yeGFP molecular brightness (see details in Additional file 1), obtained from the analysis of sN&B data similar to (and including) data shown in **A**. The raw quantitative data underlying **B** are provided in Additional file 1

Asynchronous log-phase cultures were imaged using scanning Number and Brightness (sN&B) microscopy [11, 24]. WT and Whi5-PP7tag cells in the absence of yeGFP did not exhibit any fluorescence beyond auto-fluorescent background (Additional file 2: Videos S1 and Additional file 4: Video S3, and Fig. 1A, left), whereas clear yeGFP expression was observed in yeGFP and Whi5mRNA-24x-yeGFP cells (Additional file 3: Videos S2 and Additional file 5: Video S4 and Fig. 1A, right). Bright and highly mobile *WHI5* mRNA spots were observed only in Whi5mRNA-24x-yeGFP cells, i.e. in presence of both the PP7 RNA hairpin at the C-terminal of *WHI5* mRNA, and the 2x-yeGFP-tagged PP7 coat protein (Additional file 5: Video S4). Strikingly, *WHI5* mRNA spots were observed in cells at all cell cycle stages, as determined by their size and/or budding pattern (Additional file 5: Video S4).

We next computed the cell-averaged brightness from this sN&B data using our custom analysis software [11]. The measured fluorescence brightness was 0.29 in Whi5-PP7tag cells (no yeGFP, background brightness), 0.39 in yeGFP cells expressing the PP7 coat protein-2x-yeGFP construct, and 0.78 in Whi5mRNA-24x-yeGFP cells (Fig. 1B). We note that strong background brightness could possibly stem from yeGFP photobleaching upon repeated laser exposure. From this data we computed the background-corrected brightness of 2x-yeGFP as the average brightness of yeGFP cells minus the background brightness (0.39–0.29 = 0.1), and the background-corrected brightness of Whi5mRNA-24x-yeGFP to 0.78–0.29 = 0.49, corresponding to a 2x-yeGFP mean stoichiometry of 0.49/0.1 = 4.9 (yeGFP stoichiometry of 9.8). Hence, the presence of 12 copies of the PP7-specific RNA hairpin on *WHI5* mRNA resulted in the expected dramatic (~ fivefold) increase in 2x-yeGFP oligomerization, strengthening the conclusion that the observed spots reveal *WHI5* mRNA molecules, not auto-fluorescent artefacts.

Despite its high sensitivity and ability to characterize the molecular nature of the bright spots in Whi5mRNA-24x-yeGFP cells, sN&B required a small laser excitation volume and strong confocal conditions, resulting in a depth of field < 0.8 µm. Hence, sN&B only imaged a cross section of the cells, not their entire volume, and Z-stacks acquisition in sN&B mode would result in rapid photobleaching. Hence sN&B was inadequate to accurately count *WHI5* mRNA spots in individual cells.

In this purpose, we hence resorted to standard confocal imaging of Whi5mRNA-24x-yeGFP sample cells, and of yeGFP cells as a negative control. The imaging parameters were optimized to (1) collect light from the entire cell volume to capture all *WHI5* mRNA molecules, (2) minimize imaging time to prevent double counting and (3) increase the signal-to-noise ratio to avoid missing detections (see Methods). We imaged thousands of cells from 15 to 20 fields of view (FOVs). Data were analyzed using the trainable WEKA segmentation plugin in ImageJ [25]. yeGFP negative control cells were used alongside with *WHI5* mRNA-tagged sample cells during WEKA training to ensure that *WHI5* mRNA-independent yeGFP spatial fluctuations and random clustering did not yield false detections. WEKA-segmented *WHI5* mRNA spots were then manually scored and correlated with the cell cycle stage as determined by the budding pattern (see Methods).

**Fig. 2:**
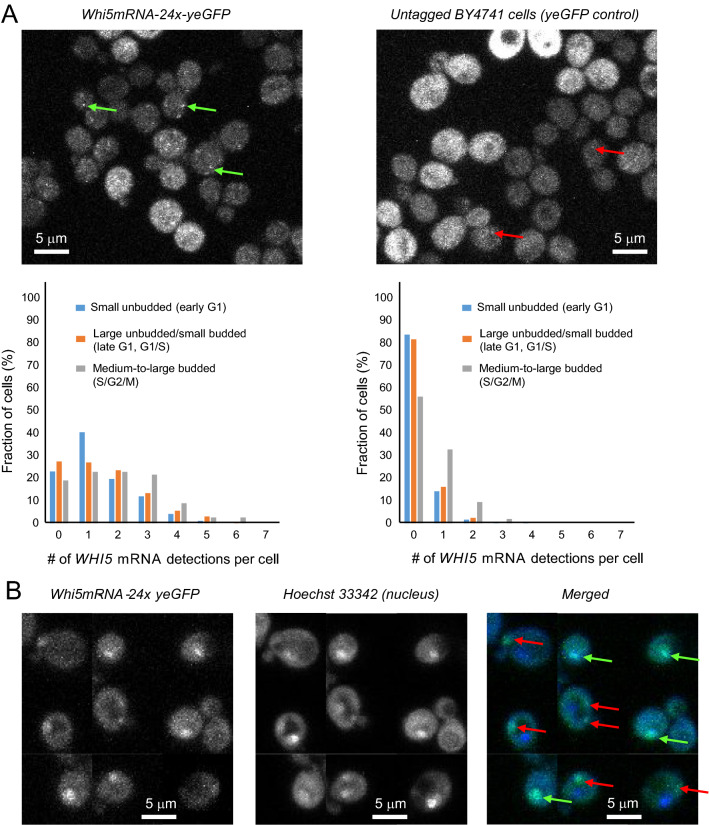
*WHI5* mRNA is expressed in similar amount across all cell cycle stages. **A** Top: example images of Whi5mRNA-24x-yeGFP (sample) cells (left), and control yeGFP cells (right). Scale bar: 5 µm. Examples of *putative WHI5* mRNA spots and artifactual yeGFP bright spots are indicated with green and red arrows respectively. Bottom: distribution of the number of *WHI5* mRNA detections per cell as determined by confocal microscopy in small unbudded (blue), large unbudded/small-budded (orange) and medium-to-large budded (grey) Whi5mRNA-24x-yeGFP cells (left, N = 488, 435, 79 cells in the 3 cell cycle stages respectively), and yeGFP control cells (right, N = 445, 416, 107 cells in the 3 cell cycle stages respectively). **B** Example images showing *WHI5* mRNA molecules localization in single cells (left) relative to the nucleus, as determined by Hoechst 33342 staining (middle, merged images: right). *WHI5* mRNA molecules were found both in the cytosol (red arrows) and the nucleus (green arrows). Images are representative of hundreds of similar cells. Scale bar: 5 µm. The larger size of some *WHI5* mRNA spots in these experiments may be attributable to the longer time lag between the imaging of two z-stack planes (due to the exposure time of the Hoechst 33342 dye), during which mRNA molecules diffuse, to a higher excitation power, or to the presence of several *WHI5* mRNA molecules within the same nuclei. The raw quantitative data underlying **A** are provided in Additional file 1

We detected 0–5 *WHI5* mRNA spots in Whi5mRNA-24x-yeGFP cells at all cell cycle stages (Fig. 2A, left), in line with smFISH data-based quantification [19, 20]. We also scored 0–2 detections in the yeGFP negative control cells, most often 0 (Fig. 2A, right). The average number of such false *WHI5* mRNA detections was 0.20, 0.21 and 0.57 in small unbudded, large unbudded/small budded and medium-to-large budded (control) cells respectively. The false detections-corrected average numbers of *WHI5* mRNA molecules in sample cells were 1.17, 1.33 and 1.39, corresponding to a very slight ~ 15% increase in *WHI5* mRNA in large unbudded and small-budded cells compared to small unbudded, in qualitative agreement with [20]. *WHI5* mRNA localized both to the nucleus and cytosol (Fig. 2B).

In summary, single live cell *WHI5* mRNA quantification correlated closely the results obtained in fixed cells or at population level in [19, 20]. *WHI5* is thus transcribed in G1, and *WHI5* mRNA molecules are found in very similar amounts throughout the cell cycle.

### Methods

#### Strains construction

All strains were built in the BY4741 background using LiAc transformation. We PCR-amplified the 12xPP7-KanMX tag sequence from the pDZ617-pKAN-12xPP7-V4 plasmid (Addgene, plasmid #72237) with homology arms for the *WHI5* C-terminal region to guide homologous recombination. The tag insertion was confirmed by colony PCR and sequencing. The resulting Whi5-PP7tag and WT control cells were next transformed with pDZ536-pURA-ADE3p-PP7-PS-2x-yeGFP (Addgene plasmid #72234), a plasmid expressing from the weak ADE3 promoter the PP7 coat protein fused to 2 yeGFP, to generate Whi5mRNA-24x-yeGFP and yeGFP strains respectively.

#### Cell culture and media

For imaging, cells were grown to saturation overnight in SC-URA + 2%glucose medium at 30C in a rotary incubator, then diluted 100-fold in fresh SC + 2%glucose medium 6 h prior to imaging, yielding log-phase cultures of OD = 0.4–0.7 at the time of imaging. For *WHI5* mRNA copy number quantification, the selective SC-URA + 2%glucose medium was also used during the 6 h pre-imaging growth phase to prevent plasmid loss and subsequent artefactual enrichment of *WHI5* mRNA-negative cells. For experiments with nuclear staining (Fig. 2B), 5 µL of a 10 mM Hoechst 33342 solution in DMSO was added to 5 mL log-phase cultures for 30 min incubation in the dark prior to imaging (final dye concentration 10 µM).

#### sN&B imaging

Cells were imaged on a ISS Alba system using the sample preparation protocol described in [11]. Imaging pads were made from the culture growth medium to prevent inadvertent nutrient shifts (see details in [26]). sN&B imaging was performed using raster scans of the same 30*30 µm (256*256 pixels) FOVs, using an excitation power of 1–2 µW at 488 nm wavelength and a 64 µs pixel dwell time.

#### Confocal imaging

Cells were imaged on a Zeiss LSM800 Airyscan confocal microscope equipped with a 63× oil objective. 78*78 µm (512*512 pixels) FOVs were excited with a 488 nm laser at 6% power and maximal speed (plane scan time in Z-stack: 521 ms). A 532 nm filter and a 741 V gain were used for yeGFP fluorescence detection. Fast-scanning was chosen to prevent double detection of the same molecules. We used a large pinhole of 1.3 Airy units to collect *WHI5* mRNA-24x-yeGFP fluorescence from large optical sections ($\sim$ 2 µm). The Hoechst 33342 dye was excited at 405 nm and 0.8% power, and dye fluorescence was detected using a 460 nm bandpass filter and 582 V detection gain. Bleed-through of the Hoechst 33342 signal in the yeGFP channel was reduced to a minimum using a 535 nm high-pass filter in the latter. To compensate for the yeGFP signal loss due to filtering, yeGFP excitation power was increased to 14% in presence of nuclear staining. The same FOVs were scanned at three Z-positions (Z-stack) spaced by 1.2 µm, yielding a total imaging cross section > 4.4 µm. Using Z-stack allowed to preserve signal-to-noise ratio in each Z-plane, while still covering the entire volume of in-focus cells.

#### *WHI5* mRNA copy number quantification

Data were analyzed in ImageJ using the trainable WEKA software [25]. Z-stacks were projected using maximal intensity projection for each pixel. The resulting 2D images were segmented in WEKA using the default parameters. To train the plugin we manually segmented dozens of bright spots (Fig. 2A left green arrows, assigned to “class 1”), and other types of cell regions (“class 2”) from 5 Whi5mRNA-24x-yeGFP FOVs, but also bright spots and other types of cell regions (all assigned to “class 2” including artefactual spots, Fig. 2A right red arrows) from 5 yeGFP control FOVs. Then the remainder of both the sample and control data were automatically segmented with WEKA, resulting in a clear separation between “class 1” spots and “class 2” artefacts. The segmented “class 1” spots (“true” *WHI5* mRNA detections) were manually counted in each individual cell and associated with the cell cycle stage as visually determined by bud morphology. Out of focus cells, cells where the bud morphology could not be ascertained, and cells expressing 2x-yeGFP at a too high level preventing spot identification were removed from analysis. *WHI5* mRNA imaging experiments with nuclear staining required a larger exposure time that could confound the *WHI5* mRNA copy number quantification, and were not analyzed.

## Limitations

Even though the imaging parameters were chosen with care, we cannot formally exclude that a small number of *WHI5* mRNAs were missed or counted twice. Likewise, the random forest-based WEKA segmentation provided an unbiased way to distinguish “true” *WHI5* mRNA spots from yeGFP spatial inhomogeneities, through the inclusion of both Whi5mRNA-24x-yeGFP and yeGFP cells in training; however it is also impossible to formally exclude that true spots were missed and some fake were counted. Any such counting errors were expected to be independent of the cell cycle stage.

In order to retain the yeGFP plasmid in all cells for the quantification experiment Fig. 2, imaging was performed in selective SC-URA + 2% glucose medium. We cannot exclude that cell cycle-dependent Whi5 expression patterns were affected by the presence/absence of uracil in the growth medium. However, little if any nutrient-dependence of *WHI5* mRNA expression has been reported in smFISH experiments [20]. In addition, cells shown in Additional file 2: Video S1, Additional file 3: Video S2, Additional file 4: Video S3, Additional file 5: 4 Video S4 and Fig. 1 were imaged in SC + 2% glucose and also showed *WHI5* mRNA in similar numbers across all stages of the cell cycle. These two lines of evidence argue against uracil-driven changes in cell cycle-dependent *WHI5* mRNA expression patterns.

Finally, it is in theory possible that the *WHI5* mRNA molecules observed and quantified in G1 daughter cells were synthesized in the previous cell cycle, in which case our observations would not demonstrate *WHI5* transcription in G1. This is, however, very unlikely as the *WHI5* mRNA half-life is in the range of minutes in WT cells [22], while G1 typically lasts about an hour.

## Supplementary Information

**Additional file 1. ** Raw quantitative data underlying Figure panels 1B and 2A.

**Additional file 2: Video S1.** *WHI5* mRNA is detected across all cell cycle stages. Exemplary video showing raw sN&B imaging data (time series) from WT cells.

**Additional file 3: Video S2.** *WHI5* mRNA is detected across all cell cycle stages. Exemplary video showing raw sN&B imaging data (time series) from WT cells expressing PP7 coat protein-2x-yeGFP (yeGFP cells). The same intensity scale was chosen as for Video S1.

**Additional file 4: Video S3.** *WHI5* mRNA is detected across all cell cycle stages. Exemplary video showing raw sN&B imaging data (time series) from Whi5-PP7tag cells (not expressing yeGFP). The same intensity scale was chosen as for Video S1.

**Additional file 5: Video S4.** *WHI5* mRNA is detected across all cell cycle stages. Exemplary video showing raw sN&B imaging data (time series) from Whi5mRNA-24x-yeGFP cells. The same intensity scale was chosen as for Video S1.

## Data Availability

The imaging datasets generated and analyzed in the current study may be found in the BioImage Archive (https://www.ebi.ac.uk/bioimage-archive/) under the accession number S-BIAD491.

## Abbreviations

WT: Wild-type
SBF: Swi4/6 Cell Cycle Box-binding factor
sN&B: Scanning Number and Brightness
FOV: Field of view
smFISH: Single molecule Fluorescence In-Situ Hybridization
FACS: Fluorescence-Activated Cell Sorting
